# Comparative Analysis of *Bursaphelenchus xylophilus* Secretome Under *Pinus pinaster* and *P. pinea* Stimuli

**DOI:** 10.3389/fpls.2021.668064

**Published:** 2021-05-11

**Authors:** Hugo Silva, Sandra I. Anjo, Bruno Manadas, Isabel Abrantes, Luís Fonseca, Joana M. S. Cardoso

**Affiliations:** ^1^University of Coimbra, Centre for Functional Ecology, Department of Life Sciences, Coimbra, Portugal; ^2^CNC – Center for Neuroscience and Cell Biology, University of Coimbra, Coimbra, Portugal

**Keywords:** pinewood nematode, pine trees, pine wilt disease, plant–nematode interactions, proteomics, SWATH-MS

## Abstract

The pinewood nematode (PWN), *Bursaphelenchus xylophilus*, the pine wilt disease’s (PWD) causal agent, is a migratory endoparasitic nematode skilled to feed on pine tissues and on fungi that colonize the trees. In order to study *B. xylophilus* secretomes under the stimulus of pine species with different susceptibilities to disease, nematodes were exposed to aqueous pine extracts from *Pinus pinaster* (high-susceptible host) and *P. pinea* (low-susceptible host). Sequential windowed acquisition of all theoretical mass spectra (SWATH-MS) was used to determine relative changes in protein amounts between *B. xylophilus* secretions, and a total of 776 secreted proteins were quantified in both secretomes. From these, 22 proteins were found increased in the *B. xylophilus* secretome under the *P. pinaster* stimulus and 501 proteins increased under the *P. pinea* stimulus. Functional analyses of the 22 proteins found increased in the *P. pinaster* stimulus showed that proteins with peptidase, hydrolase, and antioxidant activities were the most represented. On the other hand, gene ontology (GO) enrichment analysis of the 501 proteins increased under the *P. pinea* stimulus revealed an enrichment of proteins with binding activity. The differences detected in the secretomes highlighted the diverse responses from the nematode to overcome host defenses with different susceptibilities and provide new clues on the mechanism behind the pathogenicity of this plant-parasitic nematode. Proteomic data are available *via* ProteomeXchange with identifier PXD024011.

## Introduction

The pinewood nematode (PWN), *Bursaphelenchus xylophilus*, is the causal agent of the pine wilt disease (PWD). It is present in its native region, North America, and also in Japan, China, South Korea, Taiwan, Portugal, and Spain. In non-native regions, this nematode has caused heavy economic losses and dramatic and irreparable changes to the native forest ecosystems (Akbulut et al., 2015). Its main hosts belong to the genus *Pinus* and it is known that there is a variation in the susceptibility of several pine species to PWN infection. The maritime pine, *Pinus pinaster*, is considered one of the most susceptible hosts, and on the other hand, the stone pine, *P. pinea*, is a low-susceptible host (Evans et al., 1996). These two species are the most representative and economically important pine species in Portugal. Differences in the host susceptibility have been validated by pathogenicity studies with artificial nematode inoculation using pine seedlings, under laboratory conditions (Kanzaki et al., 2011; Nunes Da Silva et al., 2015; Menéndez-Gutiérrez et al., 2017). Intermediate and resistant host trees can control the PWN invasion more successfully, avoiding the nematode migration through the tree or the destruction of the tissues (cortex, phloem, cambium, and resin canals) (Fukuda, 1997). On susceptible trees, the PWN begins to feed on parenchymal cells, using the resin canals to spread quickly from the entry point throughout the tree. This leads to tracheid cavitation and disruption of water transportation, which causes the appearance of wilting symptoms and tree death within a few months after the infection. The PWN can also feed on fungi that colonize dying or dead trees (Jones et al., 2008). When feeding on plants and fungi, the PWN uses the stylet to pierce the cell wall, secrete proteins, and ingest the nutrients. Additionally, PWN proteins are also secreted from other nematode natural openings. Secreted proteins are known to have important roles on nematode–plant interaction as they are involved in cell wall degradation, cellular metabolism, cellular regulation, and host-defense evasion (Shinya et al., 2013b).

Despite the great importance that PWD denotes, due to enormous economic and ecological losses, the pathogenicity mechanism of PWN is not completed understood. Advances have been made on this mainly supported by *B. xylophilus* transcriptomic and genomic studies (Kikuchi et al., 2011; Kang et al., 2012; Espada et al., 2016; Tsai et al., 2016; Tanaka et al., 2019). All these molecular data were very useful and provided the basis for the development of proteomic studies. Few studies focus principally on the nematode secretome and have been reported and allowed the identification of proteins that are actually produced and secreted. Essentially, groups of secreted proteins such as cell-wall-degrading enzymes and peptidases were identified in *B. xylophilus* secretome and associated with the plant cell wall degradation and nematode migration through host tissues. Additionally, proteins related to tolerance against host defenses such as proteins with antioxidant and detoxifying activity, proteases, and protease inhibitors were also identified (Shinya et al., 2013a; Cardoso et al., 2016). In the comparative proteomic analysis of *B. xylophilus* secretome with the secretome of the closest phylogenetic related but non-pathogenic species, *B. mucronatus*, differences in the amount of some of these secreted proteins were found, mostly with an increase of proteins with peptidase, glycoside hydrolase, and peptidase inhibitor activities in *B. xylophilus* secretome (Cardoso et al., 2016).

In the present study, the *B. xylophilus* secretome under the stimulus of a highly susceptible host, *P. pinaster*, and a low-susceptible host, *P. pinea*, was obtained and compared to gain new insights into the molecular basis of *B. xylophilus* interaction with hosts with different susceptibilities.

## Materials and Methods

### Pine Extracts and Nematodes

Pine wood extracts were prepared from 2-year-old *P. pinaster* and *P. pinea* seedlings as previously described (Cardoso et al., 2016), and the obtained solution was used to simulate pine stimulus of high-susceptible and low-susceptible trees, respectively. Briefly, 15 g of small wood pieces from pine seedling stems were soaked in 75 ml of distilled water for 24 h at 4°C. The supernatant solution was passed through a filter paper and then centrifuged through a Vivaspin 5-kDa cutoff membrane (Sartorius Stedim). The pass-through solution containing proteins and metabolites <5 kDa was collected, refiltered in a Minisart 0.2-μm cellulose acetate membrane, and used to stimulate the nematode protein secretion, simulating, *in vitro*, the natural pine stimulus. Nematodes from a Portuguese isolate (BxPt17AS) maintained in cultures of *Botrytis cinerea* grown on malt extract agar medium at 25°C for approximately 2 weeks were used. Mixed developmental nematode stages were collected with water from fungal cultures using a 20-μm sieve and washed at least three times with sterile water.

### Pine Extract Stimuli Assay

About 1 × 10^6^ nematodes were resuspended with 5 ml of pine extract previously prepared and incubated overnight at 25°C in 10 cm Petri dishes. Six biological replicates for each stimulus were performed. Nematodes were then sedimented by centrifugation and separated from the supernatant containing the secreted proteins (±5 ml). Samples containing the secreted proteins were stored at −80°C until the proteomic analysis.

### Sample Preparation for Proteomic Analysis

For the preparation of secreted proteins, an internal standard [(IS—the recombinant protein maltose-binding protein fused with green fluorescent protein (MBP-GFP)] was added in equal amounts (1 μg of recombinant protein) to each sample (Anjo et al., 2019), and the supernatants with the secreted proteins were completely dried under vacuum using a Speedvac Concentrator Plus (Eppendorf). The resulting pellets were resuspended in SDS-sample buffer, aided by steps of ultrasonication (using a 750-W Ultrasonic processor) and denaturation at 95°C. In addition to the individual replicates (in a total of six replicates per condition), two pooled samples were created for protein identification and library creation by combining one-sixth of each replicate. To improve the protein identification and create a more comprehensive library of proteins from nematodes, pooled samples of the stimulated nematodes were also digested and analyzed. Afterward, samples were alkylated with acrylamide addiction, and gel digestion was accomplished by the short-GeLC approach (Anjo et al., 2015).

### Protein Quantification by Sequential Windowed Acquisition of All Theoretical Mass Spectra

All samples were analyzed using the Triple TOF^TM^ 6600 System (ABSciex^®^) using two acquisition modes: (i) the pooled samples were analyzed by information-dependent acquisition (IDA) and (ii) the individual samples by the sequential windowed acquisition of all theoretical mass spectra (SWATH-MS) mode. Peptides were resolved by liquid chromatography (nanoLC Ultra 2D, Eksigent^®^) on a MicroLC column ChromXP^TM^ C18CL [300 μm internal diameter (ID) × 15 cm length, 3 μm particles, 120 Å pore size, Eksigent^®^] at 5 μl/min with a multistep gradient: 0–3 min 2% mobile phase B and 3–46 min linear gradient from 2 to 30% of mobile phase B. Mobile phase A corresponds to 0.1% FA with 5% DMSO, and mobile phase B to 0.1% FA and 5% DMSO in ACN. Peptides were eluted into the mass spectrometer using an electrospray ionization source (DuoSpray^TM^ Source, ABSciex^®^) with a 25-μm ID hybrid PEEKsil/stainless steel emitter (ABSciex^®^).

For the protein identification by IDA experiments, the mass spectrometer was set to scan full spectra (350–1,250 m/z) for 250 ms, followed by up to 80 MS/MS scans (100–1,500 m/z from a dynamic accumulation time—minimum 40 ms for precursor above the intensity threshold of 1,000—in order to maintain a cycle time of 3.499 s). Candidate ions with a charge state between +2 and +5 and counts above a minimum threshold of 10 counts per second were isolated for fragmentation, and one MS/MS spectrum was collected before adding those ions to the exclusion list for 25 s (mass spectrometer operated by Analyst^®^ TF 1.7, ABSciex^®^). The rolling collision was used with a collision energy spread (CES) of 5.

For SWATH-MS-based experiments, the mass spectrometer was operated in a looped product ion mode (Gillet et al., 2012), and the same chromatographic conditions were used as in the IDA run described above. A set of 168 windows (Supplementary Table 1) of variable width (containing 1 m/z for the window overlap) was constructed, covering the precursor mass range of 350–1,250 m/z. A 50-ms survey scan (350–1,500 m/z) was acquired at the beginning of each cycle for instrument calibration, and SWATH-MS/MS spectra were collected from 100 to 1,500 m/z for 19 ms resulting in a cycle time of 3.291 s from the precursors ranging from 350 to 1,250 m/z. The collision energy for each window was determined according to the calculation for a charge +2 ion centered upon the window with variable CES, according to the window.

Peptide identification and library generation were performed with Protein Pilot software (v5.1, ABSciex^®^) with the following parameters: (i) search against the annotated *B. xylophilus* protein database obtained from Wormbase Parasite derived from BioProject PRJEA64437 (Kikuchi et al., 2011) and MBP-GFP (IS), (ii) acrylamide alkylated cysteines as fixed modification, and (iii) trypsin as digestion type. An independent false discovery rate (FDR) analysis using the target-decoy approach provided with Protein Pilot software was used to assess the quality of the identifications, and positive identifications were considered when identified proteins and peptides reached a 5% local FDR (Tang et al., 2008; Sennels et al., 2009).

Quantitative data processing was conducted using SWATH^TM^ processing plug-in for PeakView^TM^ (v2.0.01, ABSciex^®^) (Lambert et al., 2013). After retention time adjustment using the MBP-GFP peptides, up to 15 peptides, with up to five fragments each, were chosen per protein, and quantitation was attempted for all proteins in library file that were identified from the ProteinPilot^TM^ search.

Only proteins with at least one confidence peptide (FDR < 0.01) in no less than three of the six replicates per condition and with at least three transitions were considered. Peak areas of the target fragment ions (transitions) of the retained peptides were extracted across the experiments using an extracted-ion chromatogram (XIC) window of 3 min with 100 ppm XIC width.

The proteins’ levels were estimated by summing all the transitions from all the peptides for a given protein that met the criteria described above (an adaptation of Collins et al., 2013) and normalized to the levels of the internal standard of each sample. Statistical analysis was carried out to identify the differentially regulated proteins using a Mann–Whitney *U* test to the proteins that fulfill these criteria, with a *q* value of 0.05 as cutoff performed in InfernoRDN (version 1.1.5581.33355) (Polpitiya et al., 2008). Pairwise comparisons were done using the normalized protein levels.

Mass spectrometry proteomics data have been deposited to the ProteomeXchange Consortium through the PRIDE (Perez-Riverol et al., 2019) partner repository with the data set identifier PXD024011.

### Functional Annotation

Gene ontology (GO) annotations were performed using the Blast2GO 5.2.5 software (Conesa and Gotz, 2008), based on the BLAST against the non-redundant protein database NCBI and InterPro database using default settings in each step. The GO analysis was done in three different categories: the molecular function that describes the gene products’ molecular activities, the cellular component that describes where gene products are active, and the biological process describing the pathways and larger processes made up of multiple gene products’ activities. GO enrichment analysis was carried out for the proteins increased in *B. xylophilus* secretome under the *P. pinea* stimulus comparing samples against all quantified proteins, using the Blast2GO software with the statistical Fisher’s exact test associated and a *P* value of 0.05 as a cutoff.

## Results

### *Bursaphelenchus xylophilus* Secretome Profile

**FIGURE 1:**
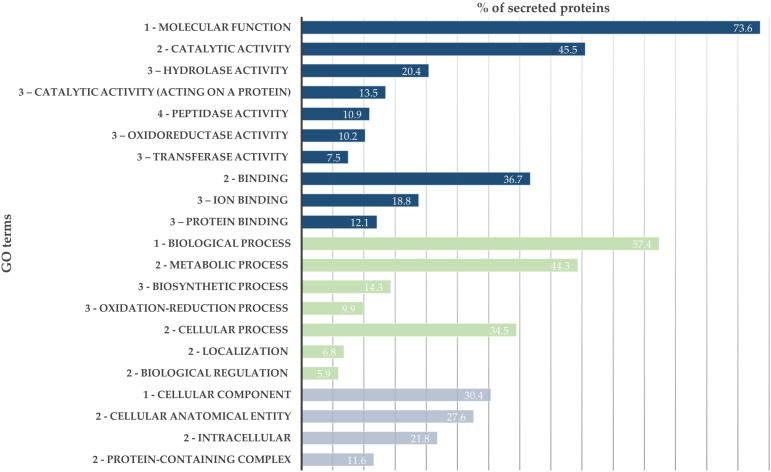
*Bursaphelenchus xylophilus* secretome profile. Distribution of 776 secreted proteins according to gene ontology (GO) terms: molecular function (blue), biological process (green), and cellular component (gray).

From the SWATH-MS analysis, 776 proteins were quantified and compared between *B. xylophilus* secretomes under the *P. pinaster* and *P. pinea* stimuli. GO analysis of the overall secretome profile showed that 73.6% of the quantified proteins were associated with a molecular function GO term, and from these, a higher percentage of proteins is associated with catalytic activity (45.5%), such as peptidase, hydrolase, or oxidoreductase activity, and binding activity (36.7%). The 57.4% of proteins associated with a biological process were mainly associated with metabolic and cellular processes. About 21.8% of identified proteins associated with cellular component GO term are intracellular proteins (Figure 1).

### Differential Quantitative Analysis of Secreted Proteins Under Different Stimuli

**FIGURE 2:**
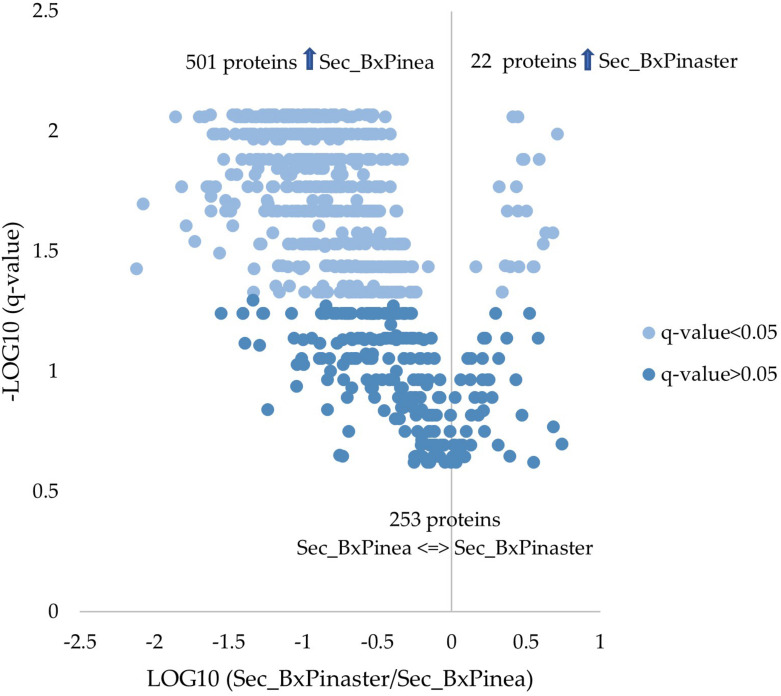
Quantitative proteomic analysis. Volcano plot with the results from the statistical analysis of the 776 proteins quantified among the secretomes of *Bursaphelenchus xylophilus* under *Pinus pinaster* (Sec_BxPinaster) and *P. pinea* (Sec_BxPinea) stimuli. Statistical analysis was performed by the Mann–Whitney *U* test, and statistical significance was considered for *q* values < 0.05.

From the 776 proteins quantified and compared between the two secretomes, 523 proteins were found differentially secreted according to the different stimuli. Twenty-two proteins were increased in *B. xylophilus* secretome under the *P. pinaster* stimulus, and 501 increased in *B. xylophilus* secretome under the *P. pinea* stimulus. The number of proteins secreted by the nematode in a higher amount was much higher when the nematode was exposed to *P. pinea* stimulus than when exposed to *P. pinaster* (Supplementary Table 2 and Figure 2). A similar tendency was also observed considering a conventional protein identification analysis of the representative pooled samples of each secretome, created to obtain the SWATH library (list of all the proteins identified in the assay) (Supplementary Figure 1).

From the 22 proteins found increased in *B. xylophilus* secretome under the *P. pinaster* stimulus, five were associated with peptidase activity, belonging to two different groups of peptidases, aspartic and serine peptidases. Proteins with hydrolase activity were also found increased: two phosphatases; a lysozyme-like protein belonging to glycoside hydrolase family 18 (GH18); a lipase EstA/esterase EstB; and a trehalase, belonging to GH37. With oxidoreductase activity, a short-chain dehydrogenase/reductase (SDR) was found increased. Six proteins are very diverse and have several different functions assigned. One of them is described as a signal recognition particle involved in binding processes. An intermediate filament protein, component of the cytoskeleton, and an integral component of the membrane were also identified. The other three proteins increased are a C-type lectin, a degenerin, and a protein involved in chromosomes’ structural maintenance. Five of them could not be annotated (Table 1).

**TABLE 1:** Description of increased proteins in *Bursaphelenchus xylophilus* secretome under the *Pinus pinaster* stimulus, based on molecular function gene ontology terms.

**Activity**	**Annotation**	**No. of proteins**	**Protein ID**
Peptidase	Aspartic peptidase	4	BXY_0035000.1
			BXY_0555800.1
			BXY_0820600.1
			BXY_0821000.1
	Serine peptidase	1	BXY_0959000.1
Hydrolase	Acid sphingomyelinase	1	BXY_0542900.1
	Histidine acid phosphatase	1	BXY_1236200.1
	Lysozyme-like protein (GH18)	1	BXY_0522000.1
	Lipase EstA/esterase EstB	1	BXY_1125700.1
	Trehalase (GH37)	1	BXY_1306200.1
Oxido reductase	Short-chain dehydrogenase/reductase	1	BXY_0328000.1
Diverse	Signal recognition particle	1	BXY_1012800.1
	Intermediate filament tail domain protein	1	BXY_1639600.1
	Integral component of membrane	1	BXY_0888500.1
	C-type lectin	1	BXY_0360300.1
	Degenerin unc-8	1	BXY_1546700.1
	Structural maintenance of chromosomes protein	1	BXY_1747100.1
	Putative proteins with no description	5	BXY_0073000.1
			BXY_1760900.1
			BXY_0799700.1
			BXY_0583800.1
			BXY_0463500.1

In order to find which group of proteins are overrepresented in increased proteins in *B. xylophilus* secretome under the *P. pinea* stimulus, a GO enrichment analysis (Fisher’s exact test and a *P* value of 0.05 as a cutoff) was done against the 776 quantified proteins. This analysis revealed a strong enrichment of proteins associated with binding activity, and from these, the most represented proteins are related to protein binding. On lower GO levels, cytoskeletal protein binding and actin binding are also terms overrepresented in increased proteins (Table 2). On the biological process category, the most represented GO term is the regulation of biological process. Many of these proteins are related to cellular catabolic processes. Proteins associated with cellular component were also found enriched, related to cytoskeleton and cell periphery (Table 2).

**TABLE 2:** Gene ontology (GO) enrichment analysis of the 501 proteins increased in *Bursaphelenchus xylophilus* secretome under the *Pinus pinea* stimulus.

**GO ID**	**GO level**	**GO name**	**GO category**	***P* value**	**No. of proteins**
GO:0005488	2	Binding	MF	2.77E-02	202
GO:0005515	3	Protein binding	MF	4.15E-02	70
GO:0008092	4	Cytoskeletal protein binding	MF	5.00E-03	12
GO:0003779	5	Actin binding	MF	5.00E-03	12
GO:0050789	2	Regulation of biological process	BP	4.24E-02	32
GO:0050794	3	Regulation of cellular process	BP	4.38E-02	27
GO:0044248	4	Cellular catabolic process	BP	4.38E-02	27
GO:0016043	4	Cellular component organization	BP	4.33E-02	22
GO:0009057	5	Macromolecule catabolic process	BP	4.81E-02	19
GO:0006996	5	Organelle organization	BP	2.86E-02	18
GO:0044265	5	Cellular macromolecule catabolic process	BP	3.89E-02	17
GO:0051603	6	Proteolysis involved in cellular protein catabolic process	BP	3.89E-02	17
GO:0044257	6	Cellular protein catabolic process	BP	3.89E-02	17
GO:0051186	4	Cofactor metabolic process	BP	1.82E-02	13
GO:0006732	5	Coenzyme metabolic process	BP	1.22E-02	10
GO:0051188	5	Cofactor biosynthetic process	BP	1.90E-02	9
GO:0007010	6	Cytoskeleton organization	BP	1.90E-02	9
GO:0009108	6	Coenzyme biosynthetic process	BP	1.90E-02	9
GO:0030029	3	Actin filament-based process	BP	2.96E-02	8
GO:0030036	4	Actin cytoskeleton organization	BP	2.96E-02	8
GO:0005856	5	Cytoskeleton	CC	7.82E-03	22
GO:0071944	3	Cell periphery	CC	2.96E-02	8

*MF, molecular function; BP, biological process; CC, cellular component. Enrichment analysis was performed against the 776 quantified proteins using the statistical Fisher’s exact test and the *P* value of 0.05 as a cutoff.*

## Discussion

The proteomic analysis carried out in this study allowed the quantification of 776 secreted proteins by *B. xylophilus* when exposed to stimuli of a highly susceptible host, *P. pinaster*, and a low-susceptible host, *P. pinea*. The functional analysis of these proteins displayed a GO distribution with a higher percentage of proteins associated with binding and catalytic activities in molecular function GO term and cellular and metabolic processes in biological process GO category, similar to the distribution previously described for *B. xylophilus* secretomes (Shinya et al., 2013a; Cardoso et al., 2016).

Interestingly, some differences were found in the distribution of proteins associated with catalytic activity compared with that previously reported for the *B. xylophilus* secretome under *P. pinaster* extract (Cardoso et al., 2016), with a higher percentage of proteins associated with hydrolase activity than with peptidase activity. Moreover, a higher percentage of proteins associated with transferase activity was found. These may reflect the *P. pinea* stimulus influence on *B. xylophilus* secretome profile obtained in this study.

The comparative quantitative data on *B. xylophilus* secretome under the different stimuli showed that 22 proteins mostly associated with nematode feeding and migration during its phytophagous phase were increased under the *P. pinaster* stimulus. From these, proteins with peptidase and hydrolase activities were the most represented. Aspartic peptidases are described predominantly in functions associated with the digestion of nutrients (Malagón et al., 2013), and several aspartic peptidases have been found on *B. xylophilus*, including on the nematode secretome. Shinya et al. (2013a) mentioned that a large number of aspartic peptidases are expressed in *B. xylophilus*, and Cardoso et al. (2016) reported that five aspartic peptidases have an increased expression when compared with *B. mucronatus*, a related but not pathogenic nematode species. Recently, Cardoso et al. (2019) studied the transcript levels of three aspartic peptidases when stimulated with *P. pinaster* extract and when stimulated with *P. pinea* extract, the same species used in this work, obtaining higher levels of transcripts of these proteins when exposed to *P. pinaster* extract. These discoveries are in line with our findings, where the family of peptidases was found increased in the secretome under the stimulus of the high-susceptible host, *P. pinaster*. In addition to the aspartic peptidases, a serine peptidase was also found increased in the *P. pinaster* stimulus. This family of peptidases is believed to be related to the invasion of host tissues, being very important for that process to occur (Sakanari and Mckerrow, 1990).

From the five proteins identified with hydrolase activity, two are phosphatases, one is a histidine acid phosphatase, and the other an acid sphingomyelinase. To date, *in vivo* functions of the phosphatases are not well defined, and the histidine acid phosphatase is the one with more information available for nematodes. The histidine acid phosphatase belongs to a wide class of high molecular weight phosphatases with an acid ideal pH that usually cleaves phosphomonoester substrates (Fukushige et al., 2005). The remaining three proteins with hydrolase activity are a trehalase, a lipase EstA/esterase EstB family protein, and a lysozyme-like protein. The protein identified as a trehalase belongs to the GH37 family and is an enzyme that hydrolyses the disaccharide trehalose into two molecules of D-glucose (Łopieńska-Biernat et al., 2019). Trehalose is important in nematode physiology as an energy source and as a protection agent against environmental stress (Pellerone et al., 2003). The influence of trehalose against environmental stresses like desiccation and freezing is known in nematodes. Trehalose interacts with lipid membranes and proteins to protect them from damage caused by those stresses (Behm, 1997). The increased amount of trehalase can be the nematode’s response to a less aggressive environment that allows the nematode to dismiss trehalose as a protective agent and use it as an energy source. Lysozymes are enzymes that cleave peptidoglycan, a vital constituent of the bacteria cell wall, and may have a role in nematode protection against pathogenic bacteria (Boehnisch et al., 2011). Moreover, alongside with *B. xylophilus*, several bacterial species are associated with the nematode (Vicente et al., 2012), and the lysozyme enzymes secreted by the nematode may be involved in the restriction of bacterial growth in order to reduce the competition for food resources, as previously suggested (Espada et al., 2016). Possibly also related to PWN–bacteria interaction, a C-type lectin was found increased in the secretome under the *P. pinaster* stimulus. The C-type lectin domain has been proposed to contribute to the immune system of nematodes. Experimental evidence suggests that upon a bacterial invasion in *Caenorhabditis elegans*, this protein is involved in the immune response. They participate in cell adhesion, glycoprotein clearance, and binding of pathogen molecules. This group of proteins participates on binding of carbohydrates, namely peptidoglycan molecules (Schulenburg et al., 2008; Bauters et al., 2017), suggesting that this protein can be involved in nematode protection against pathogens, such as bacteria. These two upregulated secreted proteins can be working together, one as a binding protein capable to interact with bacteria carbohydrates and the other capable to degrade these carbohydrates, to protect the nematode and reduce the competition for food resources.

From the remaining increased secreted proteins under the *P. pinaster* stimulus, one is a SDR associated with oxidoreductase activity. This family of proteins is involved in the detoxification and excretion of compounds that are harmful to the organisms (Lindblom and Dodd, 2006), and 25 transcripts encoding SDR were found on *B. xylophilus* transcriptome (Yan et al., 2012). In order to understand the response mechanisms of *B. xylophilus* to defensive compounds produced by plants when infected by the nematode, Li et al. (2019) studied the response of the nematode when exposed to α-pinene and found that, when the nematode is exposed to this compound, some genes of several families of proteins related with detoxification process are upregulated. Among those upregulated genes, some are related to the SDR family, showing that this type of proteins is important for the nematode detoxification process.

A degenerin unc-8 protein was also found increased in *B. xylophilus* secretome under *P. pinaster*. Degenerins are known to be involved in ion channel activity and have been proposed as important for the modulation of nematode locomotion (García-Añoveros et al., 1998; Kellenberger and Schild, 2002).

Remarkably, the number of proteins secreted by the nematode in a higher amount was much higher when the nematode was exposed to a low-susceptible tree stimulus than when exposed to a highly susceptible tree. GO enrichment analysis of the increased proteins in the secretome under the *P. pinea* stimulus revealed an enrichment of proteins with binding activity, particularly the actin-binding proteins. In agreement with the actin-binding activity, several GO terms associated with the cytoskeleton and actin function were found enriched considering the biological process category. Terms like cytoskeleton organization, actin filament-based process, or actin cytoskeleton organization represent a great number of proteins more expressed under the *P. pinea* stimulus. Actin is a component of the cytoskeleton, with various functions on eukaryotic cells, and is involved in cell morphology, endocytosis and intracellular trafficking, motility, and cell division, among others. Actin activity is controlled by actin-binding proteins (Winder and Ayscough, 2005). In plant-parasitic nematodes, an actin-binding protein was described as an effector for the root knot nematode *Meloidogyne incognita*, being secreted by the nematode to the host plant, interfering in actin functions and promoting the parasitism (Leelarasamee et al., 2018).

The differences found in *B. xylophilus* secretome under the *P. pinaster* and *P. pinea* stimuli revealed a clear different response of the nematode to these two hosts with different susceptibilities. A much higher number and type of proteins were found increased in the nematode secretome when stimulated with the less susceptible host stimulus representing a clear response to a more challenging environment, *P. pinea*. This could be a consequence of that difficult environment, leading to a more intense production and secretion of proteins to overcome the plant host defenses.

## Data Availability Statement

The datasets presented in this study can be found in online repositories. The names of the repository/repositories and accession number(s) can be found below: https://www.ebi.ac.uk/pride/archive/projects/PXD024011.

## Author Contributions

SA, BM, IA, LF, and JC conceived and designed the experiments and revised and edited the manuscript. HS, SA, and JC performed the experiments and analyzed the data. HS wrote the original draft. All authors have read and approved the final version of the manuscript.

## Conflict of Interest

The authors declare that the research was conducted in the absence of any commercial or financial relationships that could be construed as a potential conflict of interest.

## Supplementary Material

Supplementary Figure 1 Venn diagram comparing the proteins identified in each pooled sample used to obtain the SWATH-MS library. Each pool was created by combining one-sixth of each individual biological replicate and was analyzed in data dependent acquisition mode for protein identification and library generation. A total of 324 proteins (22% of all the identified proteins) were common to the secretomes of *Bursaphelenchus xylophilus* under *Pinus pinaster* (Sec_BxPinaster) and *P. pinea* (Sec_BxPinea) stimuli.

Supplementary Table 1 SWATH-MS method.

Supplementary Table 2 Secretome analysis by SWATH-MS. Statistical analysis was performed by Mann–Whitney *U* test and statistical significance was considered for q-values <0.05. Pairwise comparisons were performed using the normalized protein levels subjected to Log10 transformation. The levels of the proteins were estimated by summing all the filtered transitions from all the filtered peptides for a given protein and normalized to the total intensity within the same experimental condition.
