# Supplementary material for: Comparative Analysis of Bursaphelenchus xylophilus Secretome Under Pinus pinaster and P. pinea Stimuli
Source: Front Plant Sci. 2021 May 11;12:668064. doi: 10.3389/fpls.2021.668064 (PMC8144518; doi:10.3389/fpls.2021.668064)
Supplement: Supplementary Figure 1 — Venn diagram comparing the proteins identified in each pooled sample used to obtain the SWATH-MS library. Each pool was created by combining one-sixth of each individual biological replicate and was analyzed in data dependent acquisition mode for protein identification and library generation. A total of 324 proteins (22% of all the identified proteins) were common to the secretomes of Bursaphelenchus xylophilus under Pinus pinaster (Sec_BxPinaster) and P. pinea (Sec_BxPinea) stimuli. [file Image_1.pdf]

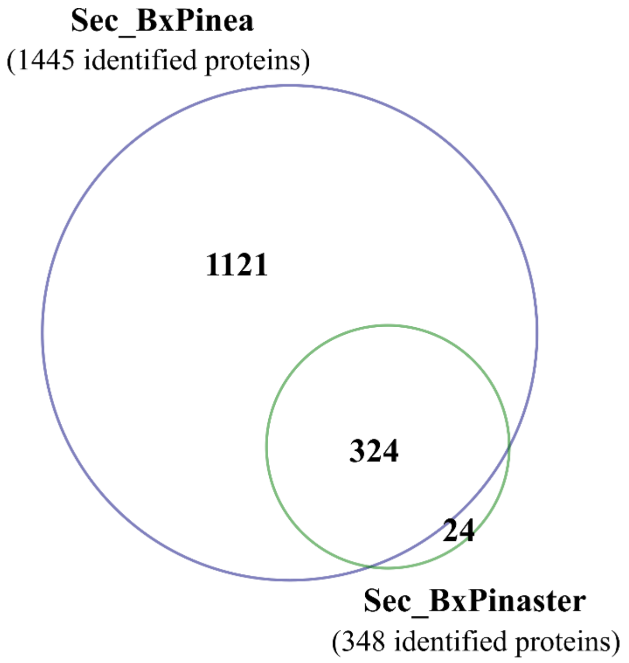

Total: 1467 identified proteins

(22% commonly identified)

Supplementary Figure S1. Venn diagram comparing the proteins identified in each pooled sample used to obtain the SWATH-MS library. Each pool was created by combining one-sixth of each individual biological replicate and was analyzed in data dependent acquisition mode for protein identification and library generation. A total of 324 proteins (22% of all the identified proteins) were common to the secretomes of *Bursaphelenchus xylophilus* under *Pinus pinaster* (Sec_BxPinaster) and *P. pinea* (Sec_BxPinea) stimuli.
